# Impact of hygiene and sanitation in ruminant slaughterhouses on the bacterial contamination of meat in Central Java Province, Indonesia

**DOI:** 10.14202/vetworld.2022.2348-2356

**Published:** 2022-09-30

**Authors:** Edy Dharma, Haryono Haryono, Aldi Salman, Pangesti Rahayu, Widagdo Sri Nugroho

**Affiliations:** 1 Postgraduate Student at Veterinary Science Study Program, Faculty of Veterinary Medicine, Universitas Gadjah Mada, Yogyakarta, Indonesia; 2 Veterinary Laboratory, Livestock and Animal Health Services Office, Central Java Province, Indonesia; 3 Department of Veterinary Public Health, Faculty of Veterinary Medicine, Universitas Gadjah Mada, Indonesia

**Keywords:** bacterial contamination, hygiene sanitation, meat, slaughterhouse

## Abstract

**Background and Aim:**

Ruminant slaughterhouse is one of the food-producing units to meet the protein demand of the people in Central Java. This study aimed to evaluate the implementation of sanitation and hygiene in ruminant slaughterhouses in Central Java based on their veterinary control number(NKV) certification and the microbiological quality of the meat produced.

**Materials and Methods:**

This study was conducted from September 2021 to December 2021. Thirty-three priority slaughterhouses, representing 33 districts/cities in Central Java Province, were assessed for their hygiene and sanitation practices according to the NKV criteria mandated by The Minister of Agriculture Regulation No. 11/2020 on NKV Certification for Animal Production Unit. Sixty-six meat samples from these slaughterhouses were obtained for microbiological analysis. The total plate count (TPC), counts of *Escherichia coli* and *Staphylococcus aureus*, and the presence of *Salmonella* spp. were determined. The microbiological tests followed the standard national testing procedure according to the Indonesian National Standard 2897:2008 on Method of Analysis for Microbiological Contaminants in Meat, Eggs, Milk, and its derived products.

**Results:**

The sanitation hygiene assessment of the 33 slaughterhouses showed that seven (21.2%) met the NKV criteria level 3, while the others did not. The average TPC of the meat samples was 1.57 × 10^5^ CFU/g (4.93 log_10_), the *S. aureus* count was 7.6 CFU/g, and the *E. coli* count was 9.2 most probable number/g. Only one sample (1.50%) tested positive for *Salmonella* spp. A comprehensive assessment comparing the NKV criteria with the level of meat contamination showed that the ruminant slaughterhouses that satisfied the NKV criteria had more meat samples (85.71%), on average, that complied with the Indonesian National Standard for microbial contamination compared with those that did not satisfy the NKV criteria (69.23%). The odds ratio was 2.67.

**Conclusion:**

Most of the priority ruminant slaughterhouses in Central Java did not meet the NKV standards. The research only looks at the level of hygiene sanitation according to NKV standards in slaughterhouses, the level of contamination produced does not reflect the level of the consumer; therefore, the level of contamination should continue to be investigated at the post-production stage.

## Introduction

Meat contains proteins, essential amino acids, fats and fatty acids, carbohydrates, vitamins, and minerals, all of which help in cell maintenance and repair and provide energy for daily activities [1, 2]. This nutritional composition, however, also provides the best medium for the growth of spoilage microbes and foodborne pathogens. The ability of microorganisms to attach to surfaces where meat is stored while being sold often causes meat contamination [3]. Poor meat quality can cause foodborne illnesses, such as those caused by contaminating *Escherichia coli* O157 H7, *Salmonella* spp., *Campylobacter* spp., *Yersinia enterocolitica*, and *Listeria monocytogenes*. The level of *E. coli* contamination is used to assess food microbiological quality and determine whether proper sanitation has been maintained [4]. *Escherichia coli* is used as an indicator for detecting fecal contamination in drinking water and other matrices [5]. *Staphylococcus aureus*, a commensal and opportunistic pathogen, causes one of the most harmful types of food poisoning in the world. *Staphylococcus aureus* can cause a broad spectrum of infections, ranging from superficial skin conditions to severely invasive diseases [6]. Common symptoms of foodborne illnesses include vomiting, diarrhea (with or without blood), fever, abdominal cramping, headache, dehydration, myalgia, and arthralgia [7]. Bacterial contamination of meat can occur in slaughterhouses until it is ready for consumption. In general, contamination occurs when the meat comes into contact with dirty hands, clothing, equipment, or facilities. Keeping the processing areas clean will result in lower chances of microbial cross-contamination [8]. Under optimum conditions, bacterial cells can double every 15–30 min. For most bacteria, individual cells can multiply into more than a million in 5 h [9].

The application of the slaughter process also impacts microbial contamination, affecting meat quality and food safety for consumers [10]. High levels of microbial contamination in meat reduce its shelf life and adversely affect its sensory properties [8]. In Indonesia, the quality assessment of the sanitation and hygiene of slaughterhouses is achieved by evaluation and categorization based on standards set by the government. To recognize the fulfillment of sanitary and hygiene requirements in slaughterhouses, such as good veterinary practices, biosecurity, animal welfare, buildings, facilities and equipment, meat handling, hygiene personnel, sanitary hygiene, and testing by the Accredited External Laboratory, certificates of Veterinary Control Number (NKV) are issued in accordance with The Minister of Agriculture Regulation No.11 of 2020 [11]. Central Java is a province that produces 59.952 tons of beef per year [12], most of which comes from 82 ruminant slaughterhouses (rumah potong hewan ruminansia [RPH-R]) owned by the government in districts and cities that engage in ruminant livestock slaughter to meet the needs of local communities [13]. The impact of sanitation hygiene applications on the microbial contamination of meat produced in RPH-R in Central Java needs to be evaluated.

This study aimed to determine the level of hygiene sanitation applications in government-owned RPH-R in Central Java and the level of microbial contamination, including the total plate count (TPC), the amount of *E. coli*, *S. aureus*, and *Salmonella* spp., in the meat produced from these slaughterhouses.

## Materials and Methods

### Ethical approval

Ethical approval for animal research was not required as live animals were not used in this study.

### Study period and location

This study was conducted from September 2021 to December 2021. Thirty-three priority slaughterhouses, representing 33 districts/cities in Central Java Province, were assessed for their hygiene and sanitation practices.

### Selection and NKV assessment of RPH-R

A total of 33 government slaughterhouses that slaughter bovines (cows and/or buffaloes) in Central Java Province were selected for this study, considering those with the largest number of slaughtered cattle or those that became a development priority in the city or regency. The assessment of slaughterhouses used the NKV assessment formula, which includes good veterinary practices, building facilities and equipment, personal hygiene, and slaughter and meat handling processes. The results, based on the number of major and minor mismatched findings in accordance with the NKV regulations, led to the RPH-R being grouped into four categories: level 1 (best), 2, 3, and no criteria (NC).

### Meat samples for microbiological testing

A total of 66 meat samples were collected from the 33 slaughterhouses for microbiological testing, two samples per slaughterhouse. The meat samples were obtained after skinning and the carcass was cut before being transported in a vehicle to the Veterinary Public Health Laboratory of the Central Java Provincial Animal Husbandry and Health Office, where procedures following the Indonesian National Standard (SNI) 2897:2008 were undertaken to determine the level of contamination in the meat samples using TPC, and test for *E. coli, S. aureus*, and *Salmonella* spp.

### Total plate count

The TPC was determined by initially homogenizing 25 g of each meat sample with 225 mL of 0.1% Buffered Peptone Water (BPW), which was diluted 10-fold, for 1–2 min. A serial dilution was performed by adding 1 mL of the previous suspension into 9 mL of BPW. For each dilution, 1 mL of the suspension was placed in a Petri cup, 15–20 mL of plate count agar cooled to 45°C was added, and the cup was incubated at 34–36°C for 24–48 h. The number of colonies for each dilution series, except the Petri dishes containing spreader colonies, was calculated. Plates with 25–250 colonies were selected [14].

### *Escherichia coli* count

The number of *E. coli* in the samples was estimated using the most probable number (MPN). In the hypothesis test (three series of tubes), up to 25 g of sample was weighed, homogenized in up to 225 mL (1:9) of 0.1% BPW in the stomacher, and diluted within the range of 10–1000-fold. From each dilution, 1 mL of sample was pipetted into three series of lauryl sulfate tryptose broth (LSTB) tubes containing a Durham tube within. The samples were incubated at 35°C for 24–28 h. The presence of emerging gas bubbles in the Durham tube indicated a positive test result. The resending test was not repeated. In the confirmation test, the positive strain was transferred using an inoculation needle from each LSTB tube into an *E. coli* broth (ECB) tube containing a Durham tube within. The ECB tube was incubated at 45.5°C for 24 h. When it tested negative, the tube was incubated again until 48 h. The amount of *E. coli* per gram of meat was determined using the MPN table based on the number of ECB tubes that contained gas inside the Durham tube. During isolation and identification, a positive sample from the ECB was scratched on eosin methylene blue agar and incubated at 35°C for 18–24 h. Black/dark *E. coli* colonies, 2–3 mm in diameter, were observed at the center of the colony with or without shiny metallic green color on the agar [14].

### *Staphylococcus aureus* count

*Staphylococcus aureus* was detected by inoculating 1 mL of each dilution onto Baird–Parker Agar medium, supplemented with 5% v/v egg yolk tellurite emulsion. The inoculum was flattened over the surface of the medium using a glass rod (hockey stick) and left until it was absorbed. The samples were incubated at 35°C for 45–48 h. *Staphylococcus aureus* colonies exhibited a round, slippery and smooth, and convex diameter of 2–3 mm, gray to pitch black color, and surrounding dark zones with or without a bright outer zone [14].

### Detection of *Salmonella spp*.

In this test, 25 g sample was weighed and placed in a sterile plastic bag, 225 mL of sterile lactose broth was added, the mixture was homogenized in a stomacher for 120 h, and incubated for 24 ± 2 h at 36°C ± 1°C. Then, 1 mL of the selectively enriched sample from the lactose broth was added to 10 mL of tetrathionate broth in a sterile test tube, up to 20 μL of iodine was added, and the sample was incubated at 36°C ± 1°C for 24 ± 2 h. For isolating *Salmonella*, selective media samples that had been incubated on each selective add-on medium were placed on a scratched quadrant on xylose lysine deoxycholate agar media and incubated at 36°C ± 1°C for 24 ± 2 h in the reverse position. After incubation, any typical colonies growing on the gelatin media were observed. The *Salmonella* colonies on this media were pink with or without black in the middle; some appeared large, were shiny black in the middle, or were all black [14].

### Relationship between NKV criteria and microbial contamination

The application of NKV and microbial contamination levels was analyzed descriptively by grouping the slaughterhouses based on the level of application of the NKV list. The actual number of RPH-R eligible for NKV certificate levels 1, 2, and 3 or those that were unqualified, and the average microbial contamination detected in the meat samples were noted adjacent to each other. The relationship between the two and its strength was calculated using the Chi-squared test and odds ratios.

## Results and Discussion

### Level of NKV in RPH-R

**Figure-1:**
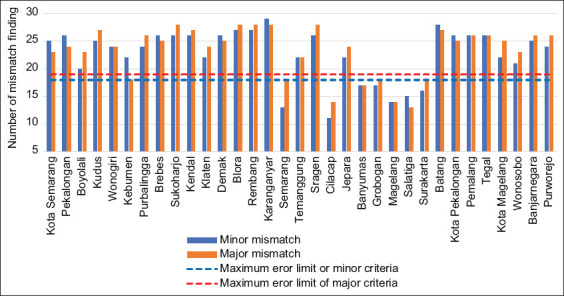
Nonconformity of priority slaughterhouses in cities/regencies.

Thirty-three priority slaughterhouses from 33 city districts were selected for this study, of which 6 RPH-R had previously obtained level 3 NKV certificates. Based on the regulations of the Ministry of Agriculture, the lowest level of NKV was obtained when the maximum error limit was 37, that is, 19 major error and 18 minor error limits (Figure-1).

**Figure-2:**
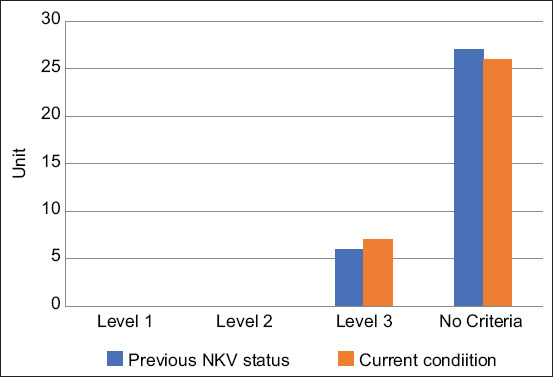
Number of slaughterhouses based on the NKV level.

An RPH-R was considered to meet the NKV criteria if the number of mismatches was below the maximum limit. Seven slaughterhouses, from Magelang, Salatiga City, Surakarta City, Banyumas, Cilacap, Semarang, and Grobogan, satisfied NKV level 3 criteria, while no slaughterhouses were included in the level 1 and 2 categories (Figure-2). The NKV certification changed in the previous year for six locations: Wonogiri, Magelang, Salatiga City, Surakarta City, Boyolali, and Banyumas. In RPH-R that met the level 3 criteria, the changes were consistent with the application of hygiene; however, the sanitation level was still low and needed regular guidance and supervision. This result is in accordance with the regulations that require level 3 slaughterhouses to be monitored and surveyed every 4 months [11].

The number of priority slaughterhouses that met the new NKV level 3 criteria showed good but low implementation levels. According to the regulations, these slaughterhouses can only supply meat to one province.

Based on the major and minor mismatched findings, RPH-R in Central Java faces the following problems:

1. Any animals that arrive are unaccompanied by a Veterinary Certificate or Animal Health Certificate
2. No physical separation exists between clean and dirty spaces
3. Light intensity in antemortem and postmortem examination area is <540 lux
4. Air flows from dirty areas to clean areas
5. Hand-washing facilities are hand-operated
6. Temperature in the carcass handling room and meat is higher than 15°C
7. Meat transport does not prevent contamination
8. No written program is available for insect control, rodents, and/or other disruptive animals
9. No laboratory testing is available to examine the effectiveness of sanitation programs.

Given these conditions, slaughterhouses need regular improvement to meet the minimum level of NKV criteria because it guarantees the maintenance of sanitary hygiene requirements. According to Kuntoro *et al*. [15], low implementation of hygiene sanitation, as indicated by a high number of mismatches on the NKV checklist, is closely related to high levels of microbial contamination in meat. Besides facilitating the supervision and monitoring of animal food products and tracking the problems related to food safety, the NKV certification of a business unit can become its identity [16].

Slaughterhouses that have met the requirements still need regular monitoring of their products because important major findings may still occur in the field. The goal is that business unit that have already earned NKV certificates guarantee the fulfillment of hygiene and sanitation requirements, assuring the safety of their animal products [17].

### Microbial contamination levels

TPC is the enumeration of microorganisms that grow in aerobic conditions at moderate temperatures of 20–45°C. This calculation encompasses all pathogens and nonpathogens and is used to determine the hygiene status of food products. The microbiological growth medium, which is a nonselective medium, is used while determining TPC [18]. High levels of microbial contamination can increase damage and decrease the shelf life of meat while potentially carrying pathogenic bacteria that can cause foodborne illnesses [8, 18].

**Figure-3:**
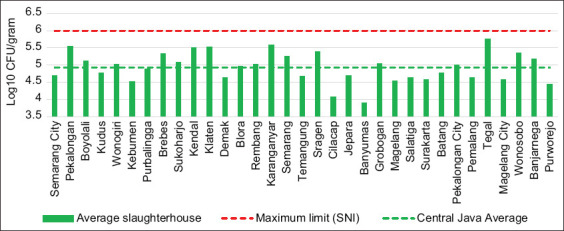
Total plate count of meat from priority slaughterhouses in each city/regency.

Figure-3 shows that the average TPC of meat from priority slaughterhouses in Central Java was 4.93, which is below the maximum limit set by SNI (1 × 10^6^ CFU/g or 6 log_10_TPC). The highest contamination value was obtained in meat from Tegal Regency with a log_10_TPC value of 5.76 while the lowest was recorded in meat from Banyumas Regency with a log_10_TPC value of 3.90. In this study, 100% of the meat samples exhibited TPC values that met the SNI standards. In contrast, Jacob *et al*. [19] showed that 63.33% of the meat samples failed to meet the SNI standards in Kupang, while Mufidah *et al*. [20] attained a value of 33.33% for the same in Probolinggo, East Java. The different levels of microbial contamination are influenced by differences in the hygiene and sanitation applications in each slaughterhouse.

Microorganisms can contaminate meat through blood circulation at the time of slaughter or due to the use of unclean equipment or improper hygiene. Contamination may also occur after slaughtering: during skinning, evisceration, carcass handling, cooling, freezing, thawing, packaging, storage, distribution, and before consumption of the meat [15]. Thus, the difference in procedures and quality of sanitation hygiene in each slaughterhouse will affect the level of contamination.

**Figure-4:**
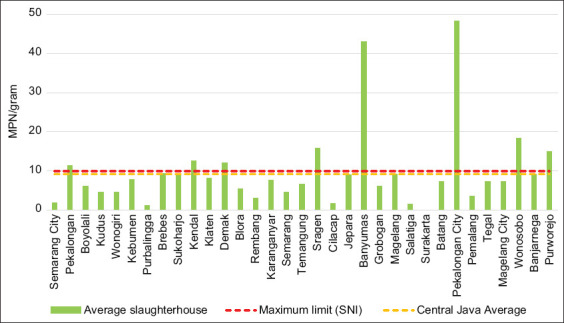
*Escherichia coli* contamination in meat from priority slaughterhouses in each city/regency.

*Escherichia coli* contamination requires special attention because a dangerous strain, *E. coli* O157:H7, which causes food and waterborne diseases, has a high prevalence of 6.3% in one of the Indonesian provinces [21]. Figure-4 shows the level of *E. coli* contamination observed in this study.

Of the 66 samples collected, 80.3% (53) were contaminated with *E. coli*. In contrast, a study conducted in East Java reported an *E. coli* contamination rate of just 32.5% [18]. A study in Africa reported a similar prevalence of 91.9% (87.2–96.0%) while another study in Malaysia found 55% of the samples contaminated with *E. coli* [22, 23]. The average contamination rate in all priority slaughterhouses in Central Java was 9.02 MPN/g, which is close to the allowed maximum limit of *E. coli* contamination (10 MPN/g) [24, 25]. Seven districts (21.21%) and 20.3% of the samples exhibited an average *E. coli* contamination exceeding the maximum allowed limit. The highest value of *E. coli* contamination (48.3 MPN/g) was recorded in the meat from Pekalongan City, while the lowest was recorded in the meat from Surakarta City (<3 MPN/g).

**Figure-5:**
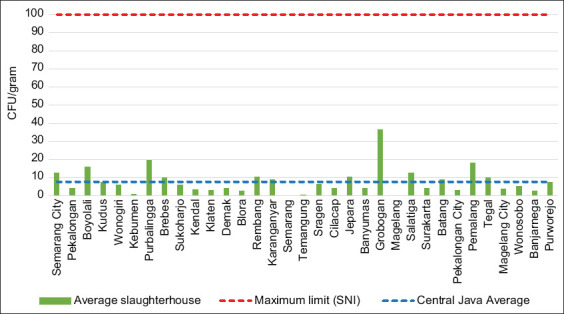
*Staphylococcus aureus* contamination in meat from priority slaughterhouses in each city/regency.

In this study, 86% of the meat samples were contaminated with *S. aureus*. However, the overall contamination rate of 7.65 CFU/g was still below the maximum limit set by SNI (100 CFU/g) [24, 25]. Figure-5 shows the level of *S. aureus* contamination observed in this study. The previous investigations in East Java, Indonesia, Ethiopia, and Malaysia recorded *S. aureus* contamination levels of 20%, 22.5%, and 32%, respectively [18, 26, 27]. Humans can be a source of contamination because we act as reservoirs by carrying the enterotoxin-producing *S. aureus* on our hands or in our nostrils, which are the two main sources of food contamination: Mechanical contact or aerosol droplets [28]. The application of hygienic food production and public education on food safety is the main strategies to prevent Staphylococcal food poisoning [29].

Globally, *Salmonella* spp. cause millions of cases of enteric diseases, and thousands of hospitalizations and deaths every year [30]. In this study, 1.5% of the samples tested positive for *Salmonella*. Similar studies from other regions have showed a *Salmonella* prevalence of 3.1%, 0%, and 2.5% [18, 31, 32]. A positive sample result is not in accordance with SNI 7388:2009, which recommends a negative result per 25 g of *Salmonella* spp. [24, 25]. *Salmonella* spp. can contaminate meat because they live in the intestines of animals. The use of sanitation systems before the slaughtered meat is cut and handled also influences *Salmonella* spp. infection [32].

### Relationship between NKV criteria and microbial contamination

Table-1 provides comprehensive details regarding the NKV criteria and the fulfillment of SNI for bacterial contamination, which was deemed fulfilled (good) if the mean microbial contamination from TPC, *E. coli*, and *S. aureus* tests was under the maximum limit of SNI and the *Salmonella* spp. test was negative.

**Table-1:** Veterinary control number (NKV) criteria and microbial contamination.

Location of Slaughterhouse	NKV criteria	Maximum contamination limit				Microbial contamination status

		TPC (10^6^ CFU/g)	*S. aureus* (10^2^ CFU/g)	*E coli* (10^1^ MPN/g)	*Salmonella*. Spp. (negative)	
Semarang City	NC	V	V	V	-	Good
Pekalongan	NC	V	V	X	-	No Good
Boyolali	NC	V	V	V	-	Good
Kudus	NC	V	V	V	-	Good
Wonogiri	NC	V	V	V	+	No Good
Kebumen	NC	V	V	V	-	Good
Purbalingga	NC	V	V	V	-	Good
Brebes	NC	V	V	V	-	Good
Sukoharjo	NC	V	V	V	-	Good
Kendal	NC	V	V	X	-	No Good
Klaten	NC	V	V	V	-	Good
Demak	NC	V	V	X	-	No Good
Blora	NC	V	V	V	-	Good
Rembang	NC	V	V	V	-	Good
Karanganyar	NC	V	V	V	-	Good
Semarang	Level 3	V	V	V	-	Good
Temanggung	NC	V	V	V	-	Good
Sragen	NC	V	V	X	-	No Good
Cilacap	Level 3	V	V	V	-	Good
Jepara	NC	V	V	V	-	Good
Banyumas	Level 3	V	V	X	-	No Good
Grobogan	Level 3	V	V	V	-	Good
Magelang	Level 3	V	V	V	-	Good
Salatiga	Level 3	V	V	V	-	Good
Surakarta	Level 3	V	V	V	-	Good
Batang	NC	V	V	V	-	Good
Pekalongan City	NC	V	V	X	-	No Good
Pemalang	NC	V	V	V	-	Good
Tegal	NC	V	V	V	-	Good
Magelang City	NC	V	V	V	-	Good
Wonosobo	NC	V	V	X	-	No Good
Banjarnegara	NC	V	V	V	-	Good
Purworejo	NC	V	V	X	-	No Good

V=under maximum limit SNI,X=upper maximum limit SNI,-= negative *Salmonella* spp.,+ = positive *Salmonella* spp.,TPC=Total plate count, Good: if the level of microbial contamination is under the maximum contamination limit according to SNI standard (comply SNI), No Good: if the level of contamination exceeds/upper the maximum contamination limit according to SNI standard (not comply SNI).

Table-2 shows the relationship between the NKV criteria and microbial contamination. Cross-tabulation showed that among the RPH-R samples that were level 3 NKV certified, 85.71% met the maximum SNI limits, while 14.3% exceeded it. Furthermore, among the samples with an NC certification, 69.23% satisfied the SNI limits, while 30.77% did not. A Chi-square value of 0.15 and an odds ratio of 2.67 with a low level of significance (p = 0.7 and p = 0.39) indicate no correlation between NKV criteria and the level of microbiological contamination. The varying microbial contamination levels of fresh meat are influenced by various factors, such as maintenance, transport, cutting and packaging, and the hygiene and processing conditions in slaughterhouses [33].

**Table-2:** Cross tabulation of NKV criteria and microbial contamination status.

	No good microbial contamination	Good microbial contamination	Total
No criteria	8	18	26
NKV Level 3	1	6	7
	9	24	33

Chi square : 0.15 (p=0.7) Odd Ratio: 48/18=2.67; P=0.39

To evaluate the findings presented above, sanitation implementation needs to be considered. Sanitation, hygiene, and implementation of good manufacturing practices aim to reduce the number of microbes to a safe and acceptable level [34]. Cleaning and disinfection programs contribute to good environmental conditions and they must be validated based on the regulations of each country [35]. The objectives of cleaning and sanitizing surfaces in contact with food are to remove the food (nutrients) that bacteria can grow on and to kill the bacteria that are already present. Cleaning/sanitizing procedures must be evaluated for their adequacy. Adherence to prescribed written procedures (inspection, swab testing, and direct observation of personnel) should be continuously monitored and records maintained to evaluate long-term compliance [36].

The slaughter and handling of meat at room temperature affects the rate of bacterial growth in all slaughterhouses. Besides cleanliness, keeping meat products cold is the second most important requirement for achieving the desired shelf life. Microorganisms multiply rapidly at high temperatures, and the development of mucus is a visual sign of microbial growth [8]. Temperature and gas composition are the main extrinsic factors that affect microbial growth [37]. Temperatures between 4.4°C and 60°C constitute the danger zone. When potentially harmful foods are left at temperatures in this range, the bacteria in and on those foods will grow rapidly [38]. Thus, the level of contamination can be reduced again if a lower temperature is applied.

Another problem in all slaughterhouses is the nonseparation between “dirty” and “clean” areas, which can lead to cross-contamination of meat products with animal manure, due to workers handling dirty areas, or with products that are unfit for consumption. Rahkio and Korkeala [39] reported associations between the microbiological contamination of air and carcasses and the movements of workers. The layout of the slaughtering line was shown to be important in decreasing airborne contamination. The separation of the clean and unclean parts of the line and that of the weighing area from other clean parts of the line decreases the contamination level. Adjusted air flow is expected to reduce carcass contamination.

This study showed that the extent of microbial contamination of the meat, in terms of the TPC and *Staphylococcus* count, still met the requirements, but contamination levels are likely to rise during transportation to consumers. Notably, the conditions of the buying and selling facilities in Central Java are inadequate, which is in accordance with Aminullah *et al*. [40], who stated that, in traditional markets where beef is sold, a temperature above room temperature, crowded populations, and the lack of water to clean equipment, all affect the number of microorganisms on the meat. Thus, when the meat reaches consumers in optimal conditions, rapid bacterial growth occurs. Therefore, the status of microbes in meat to meet consumer requirements is supported by adequate treatments that can inhibit microbial growth. The factors that affect bacterial growth are divided into two groups: Intrinsic and extrinsic. Intrinsic factors include the nutritional value of meat, water content, pH, oxidation–reduction potential, and the absence of obstruction or inhibitory substances. Extrinsic factors comprise temperature, relative humidity, absence of oxygen, and form or condition [41]. The extent of meat damage depends on the initial number of microbes–meat will spoil faster if it has a high number of initial microbes. Therefore, good hygiene during meat processing in slaughterhouses is essential to determine the quality of the final product [38, 42]. The type and number of bacteria depend on early meat contamination and specific storage conditions, which can affect the development of different decay-related microbial populations; thus, directing the type and speed of the decay [43]. Bacterial contamination during the slaughter process is a safety issue that must be considered because it affects saving time in meat production [44].

Thus, the main goal of meat processing is good meat handling, which can suppress bacterial growth factors and guarantee that the meat reaches the consumer in a good condition. Veterinary Control Number certification guides the good handling of meat and guarantees that sanitation hygiene is being implemented.

## Conclusion

We found that only 7/33 (21.21%) selected for this study satisfied the NKV criteria. The contamination level was generally low, but seven slaughterhouses offered meat with an average level of *E. coli* contamination above the maximum limit. In contrast, meat from one slaughterhouse tested positive for *Salmonella* spp. Rumah potong hewan ruminansia that met the NKV criteria showed, produced more meat with a permissible level of microbial contamination than the RPH-R that did not meet the NKV criteria. Therefore, NKV certification, testifying the application of sanitation hygiene, must be implemented in all slaughterhouses.

## Authors’ Contributions

WSN: Conceptualized and designed the study, interpretation and discussed the result, supervised the research process, and revised the manuscript. ED: Sampling coordinator, collected the data and data analysis, and drafted the manuscript. HH: Collected and tested the samples and revised the manuscript. AS: Collected and tested the samples, and revised the manuscript. PR: Collected and tested the samples and revised the manuscript. All authors have read and approved the final manuscript.
